# PGP repository: a plant phenomics and genomics data publication infrastructure

**DOI:** 10.1093/database/baw033

**Published:** 2016-04-16

**Authors:** Daniel Arend, Astrid Junker, Uwe Scholz, Danuta Schüler, Juliane Wylie, Matthias Lange

**Affiliations:** Leibniz Institute for Plant Genetics and Crop Plant Research (IPK), OT Gatersleben, Corrensstraße 3, Stadt Seeland, 06466, Gatersleben, Germany

## Abstract

Plant genomics and phenomics represents the most promising tools for accelerating yield gains and overcoming emerging crop productivity bottlenecks. However, accessing this wealth of plant diversity requires the characterization of this material using state-of-the-art genomic, phenomic and molecular technologies and the release of subsequent research data via a long-term stable, open-access portal. Although several international consortia and public resource centres offer services for plant research data management, valuable digital assets remains unpublished and thus inaccessible to the scientific community. Recently, the Leibniz Institute of Plant Genetics and Crop Plant Research and the German Plant Phenotyping Network have jointly initiated the Plant Genomics and Phenomics Research Data Repository (PGP) as infrastructure to comprehensively publish plant research data. This covers in particular cross-domain datasets that are not being published in central repositories because of its volume or unsupported data scope, like image collections from plant phenotyping and microscopy, unfinished genomes, genotyping data, visualizations of morphological plant models, data from mass spectrometry as well as software and documents.

The repository is hosted at Leibniz Institute of Plant Genetics and Crop Plant Research using e!DAL as software infrastructure and a Hierarchical Storage Management System as data archival backend. A novel developed data submission tool was made available for the consortium that features a high level of automation to lower the barriers of data publication. After an internal review process, data are published as citable digital object identifiers and a core set of technical metadata is registered at DataCite. The used e!DAL-embedded Web frontend generates for each dataset a landing page and supports an interactive exploration. PGP is registered as research data repository at BioSharing.org, re3data.org and OpenAIRE as valid EU Horizon 2020 open data archive. Above features, the programmatic interface and the support of standard metadata formats, enable PGP to fulfil the FAIR data principles—findable, accessible, interoperable, reusable.

**Database URL:** http://edal.ipk-gatersleben.de/repos/pgp/

## Introduction

The technical progress in plant genomics and phenomics enables a new quality in studying plant development and underlying mechanisms and processes (1, 2). Huge amounts of data are gathered in short time and require respective capacities for their analysis, management and storage. This development is furthermore supported by decreased costs for the use of technologies. One prominent example is the decrease of sequencing costs that result in the $1000 genome (3). In particular, metabolomics, phenomics, genotyping and next-generation sequencing are major driving forces. Discussed broadly as ‘open data’ policy, research data should in general be considered as a scientific asset. However, in practice, there is a big gap between the created and actual accessible data (4). Although this issue is addressed by service provider, a high number of datasets remain unpublished. Such service provider are:

-data journals (5), e.g. GigaScience (http://www.gigasciencejournal.com) or Nature Scientific Data (http://www.nature.com/sdata),
- international consortia that support public data management (6),
- primary data repositories such as figshare (7) and
- public databases (8), like Gramene (9) as comprehensive but specialized information system with a lot of embedded functionality for comparative functional genomics in crops.

a high number of datasets remain unpublished. This applies, e.g. to unprocessed raw data (especially in data domains for which no dedicated domain repository exists yet) or results confirming and/or deepening previous observations. Estimations suggest the ‘odds of a dataset being extant fell by 17% per year’ (10). On the other hand, a survey among authors published in the *Annals of Internal Medicine* between 2008 and 2012 found that their willingness to share their data decreased from 62% to 47% over the overserved period (11, 12). The increase in unpublished (lost and/or hidden) data contradicts the scientific self-conception, which is based on the spread and gain of knowledge among the scientific community. Potential reasons for keeping datasets unpublished might be missing archives for certain data domains, i.e. phenomics, technical barriers for researchers, unaccepted citation metrics, complex metadata schemas, laborious data documentation, privacy concerns, unsupported data volume and data exploitation priorities (13).

Publishing experimental data (even outside of scientific journal publications) has major advantages for the community and the publishing scientist him/herself. It enables to test the repeatability and reproducibility of scientific experiments and allows for drawing comparisons between datasets, given that metadata are sufficiently documented. Furthermore, it will generate a refund for the publishing scientist him/herself (14). To respond the challenge of losing ‘hidden data’ for the scientific value chain, international initiatives were established (15, 16). In this context, the DataCite consortium (17) was found to support data citation, providing means to increase the acceptance of research data as legitimate contributions to scholarly records. DataCite provides a free service to assign digital object identifiers (DOIs) (17) as globally proven standard for persistent identifiers, also enabling the registration of a minimal commonly accepted set of technical metadata for a scientific dataset. Since DOIs have already represent an accepted data citation standard (e.g. by publishers, journals and public databases), these identifiers are the preferred way for citation of datasets.

**Figure 1.**
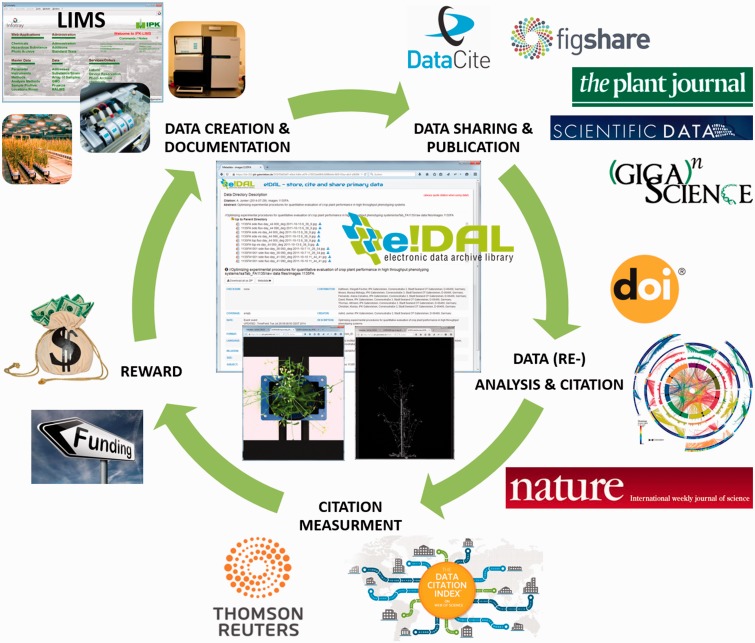
Data publication cycle. This cycle illustrates the scientific value chain for research data publication. The documentation of experimental metadata and result data represent the basis for scientific journal publications. These are the main outcomes of scientific work, representing scientific successes as the most important way of communication of research results in the community. A parallel public sharing of experimental data increase the scientific value by enabling tests for reproducibility and providing valuable resources for further downstream analysis. In turn, new findings that are reported in published datasets increase its scientific impact and boost the author’s scholarly credit. Data citation indexes are increasingly accepted as measurement for scientific success which in turn represents the most important prerequisite for project proposals and the acquisition of funding for new projects.

In this article, we introduce the Plant Phenomics and Genomics Data Publication Infrastructure (PGP) repository that implements all components of a sustainable data publication culture cycle (18) (Figure 1). The presented PGP repository contains diverse published datasets so far covering a variety of different data domains, starting with phenomics data gained in frame of high-throughput phenotyping experiments (Deutsches Pflanzen Phänotypisierungsnetzwerk (German Plant Phenotyping Network, http://www.dppn.de/dppn/en) to data gathered within Leibniz Institute of Plant Genetics and Crop Plant Research (IPK) Genome Research activities. After a brief introduction into the concept for a sustainable data publication, we describe the workflow how to publish, search and download datasets in PGP. In the last section, the current status (after 9 months of productive data publication service) and further objectives will be summarized.

## The IPK plant genomics and phenomics research data repository

Based on the universal electronic Data Archive Library (e!DAL) data publication and sharing infrastructure (19), the PGP was established in January 2015 at the IPK Gatersleben to share research data derived from studies of plant genomics and phenomics with particular emphasis on the support of association studies (integrating genomic and phenotypic information) as well as different kinds of supplement material, like drawings, source code and protocols. So far, published plant datasets cover different data domains ranging from phenotypic data, systems biology and molecular data to data from genome sequence analyses. The majority of data comprise collection of macroscopic and microscopic plant images, sequence assemblies, genotyping data, X-ray images and visual models of plant organs, raw data from mass spectrometry, paper supplemented spread sheets and software. As by December 2015, 54 datasets have been published as DOI and registered at the DataCite research data catalogue. Each dataset comprises a container including all records that are related to a particular experiment or scientific paper. The PGP repository currently hosts 21 157 data entities with an overall volume of 65.4 GB and is registered in the re3data registry (20) and the biosharing.org portal (21) as accepted data repositories. 

### Data access

**Figure 2.**
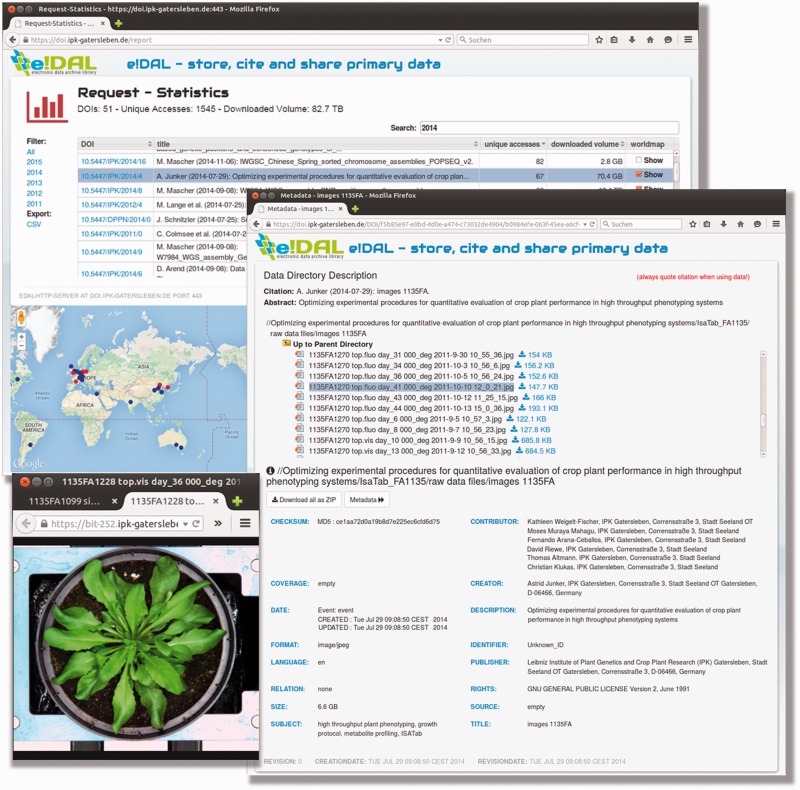
Report page PGP repository. Screenshot of the report page embedded in the PGP Repository for each experiment. The report provides information on the current data stock, access frequencies and the number of downloads for the respective DOI. All datasets are linked and access statistics are mapped on a world map.

The integrated and permanently updated report frontend (Figure 2) supports the interactive exploration of stored datasets. Beside functionalities for browsing, filtering and downloading of datasets, download and access statistics are provided for each DOI.

**Figure 3.**
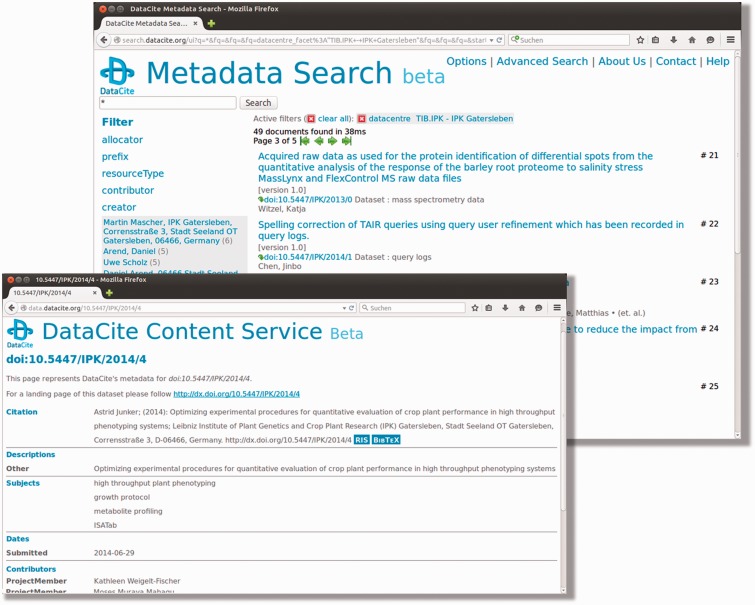
DataCite metadata search interface. This screenshot shows the web interface of DataCite, where a number of filter functionalities are implemented and additional options can be defined using the advanced search functionalities.

Using the metadata search provided by DataCite (Figure 3) (http://search.datacite.org), users are enabled to explore and search specific PGP datasets of interest. A general keyword-based search functionality over all metadata, as well as a more advanced search, allows for filtering by parameters, such as authors, dates and file types are implemented. Besides, via the provided web interface, it is also possible to use the search engine programmatically via an Apache Solr interface.

**Figure 4.**
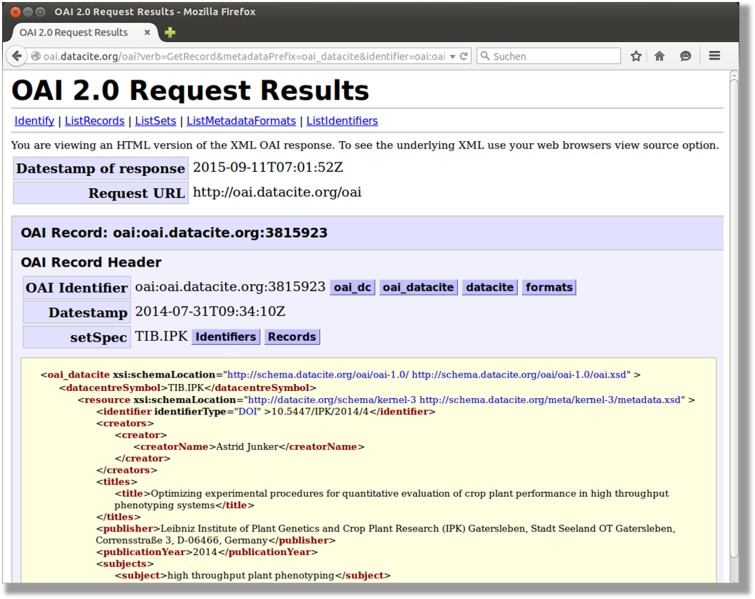
DataCite OAI-interface. This snapshot illustrates the result of an OAI request to DataCite.

In order to harvest metadata programmatically, PGP supports the Open Archives Initiative Protocol for Metadata Harvesting (OAI-PMH) (22). Because we use the DataCite interface to register DOIs and associated metadata, all required specifications are implemented. It can be used via an application programming interface (API) integrated in DataCite (http://oai.datacite.org/oai) or the OpenAire endpoint (http://api.openaire.eu/oai_pmh) (Figure 4).

### Data submission process

In order to support the reproducibility of experimental research results, it is not sufficient to just share the produced data files and make them accessible, it is rather important to guarantee that the files are readable and reusable (23). Proprietary file formats and incomplete metadata in the worst case might even prevent opening and reading of data. A sufficient data annotation and the use of standardized file formats strongly enhance the data quality.

In general, there are two types of annotations. Technical metadata are required for data processing and managing reasons and contain information like the file type, the file size or the software environment in order to guarantee a long-term readability. The number of available technical metadata schemes is relatively small. One worldwide accepted format is the Dublin Core Metadata Element Set (DCMES) (24). In contrast, semantic metadata include a wide range of more or less complex descriptions that are strongly related to a specific research field. Semantic metadata schemas are in general dynamic and reflect ongoing discussions in the related scientific community. In consequence, homogeneous experiment documentation towards a consistent and sufficient semantic annotation of research data is a still challenging task. One interesting and promising approach is the ISA-Tab format (25), which is connected with domain-specific metadata configurations. It is a general format container for semantic annotations.

Beside a sustainable data annotation to efficiently describe the performed experiments, used parameters or the implemented source code, it is necessary that the datasets and their corresponding metadata pass through an additional, internal review process before being published. In general two reviewer groups are distinguished ‘scientific’ and ‘administrative reviewers’.

Scientific evaluation: Scientific reviewers have scientific expertise in the author’s research area and are asked to evaluate the dataset for scientific quality.
Administrative evaluation: The administrative reviewers are responsible to check for publication rights (especially open access) and potential conflicts with respect to intellectual property and patent regulations of the partners/co-authors and/affiliated institutions.

To our knowledge, no defined criteria hitherto exist for a data publication review process. Therefore, we suggest a checklist including a minimal set of quality criteria for data reviewers. This prioritized guideline is based on recommendations of the nestor working group (26) and BioSharing initiative (27). The minimal set of criteria is listed below and does not represent an obligatory catalogue or checklist, but the reviewers should rather consider these criteria as recommendations supporting their decision and should reflect commonly accepted data quality standards.

Experiment documentation: Hypothesis, materials, methods, conditions, parameters and experimental factors, results.
Authorship: Ownership, license and IP issues, patents.
Data structure: Hierarchical folder structures, consistent file naming, avoid archive files.
Semantic metadata: Representation of experimental metadata using a standardized metadata scheme.

The ‘Experiment documentation’ and information about ‘authorships’ provide metadata assigned to the submitted dataset, which provides at least a minimal experimental context and dataset description as well as technical information to ensure later data access. In contrast, ‘data structure’ and ‘semantic metadata’ are no mandatory elements, but enable long-term usability and repeatability. Further scientific criteria, such as statistical validation of the dataset, are part of the data post-processing pipeline and are not obligatory for dataset publication in PGP. Although, if available, it is very valuable to complement raw data in the repository and its publication along with these, it is kept in the reasonability of the submitting scientist.

#### Submission tool

**Figure 5.**
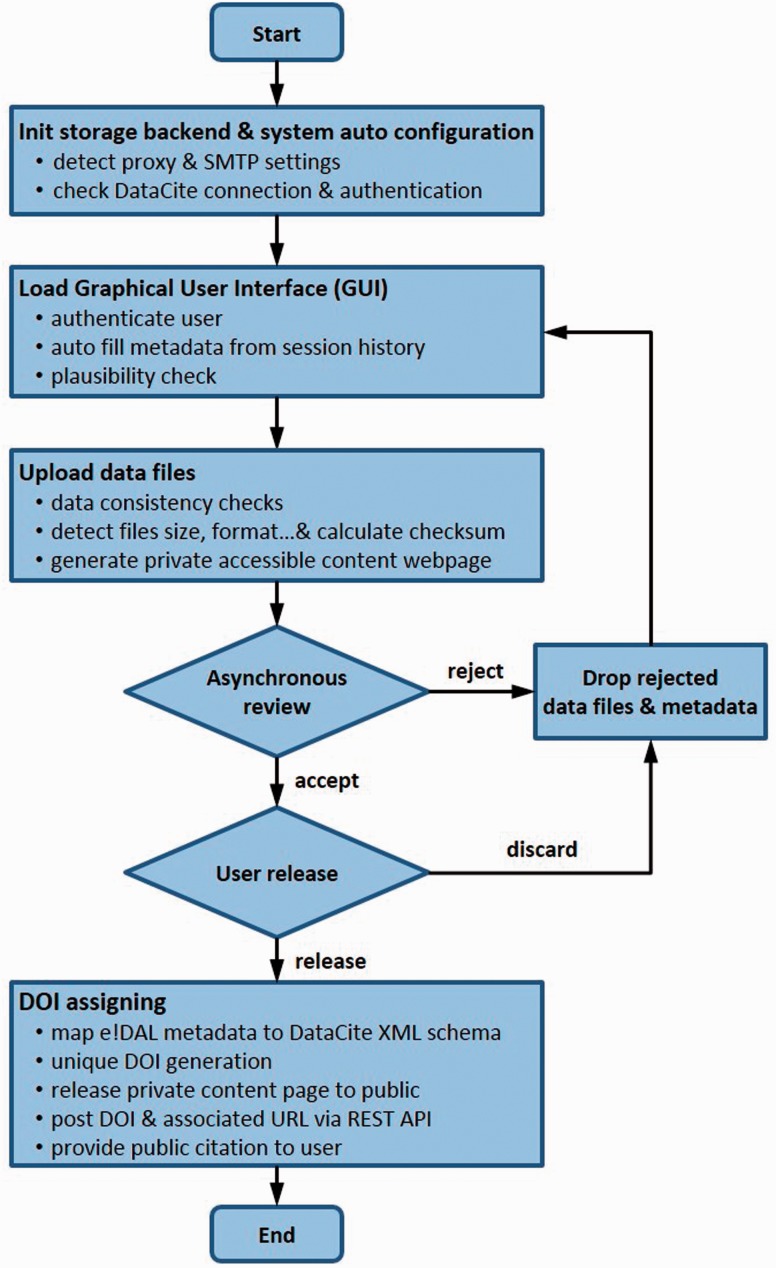
The data publication process. This flowchart illustrates the several steps of the described data publication and approval workflow.

Based on the prerequisites for data publication defined in the sections above, this section describes a software tool that meets and considers all formal and technical requirements for archiving and publishing research data and comprises all necessary functionalities from data upload to DOI registration. According to the data publication workflow (Figure 5), scientists first organize and describe their experimental data, which can then be uploaded followed by a reviewing procedure as described in Data submission process section. After approval by the reviewers, a DOI is assigned as permanent reference for data sharing. In the following, the implementation of the described workflow in the frame of a data publication tool is shown.

**Figure 6.**
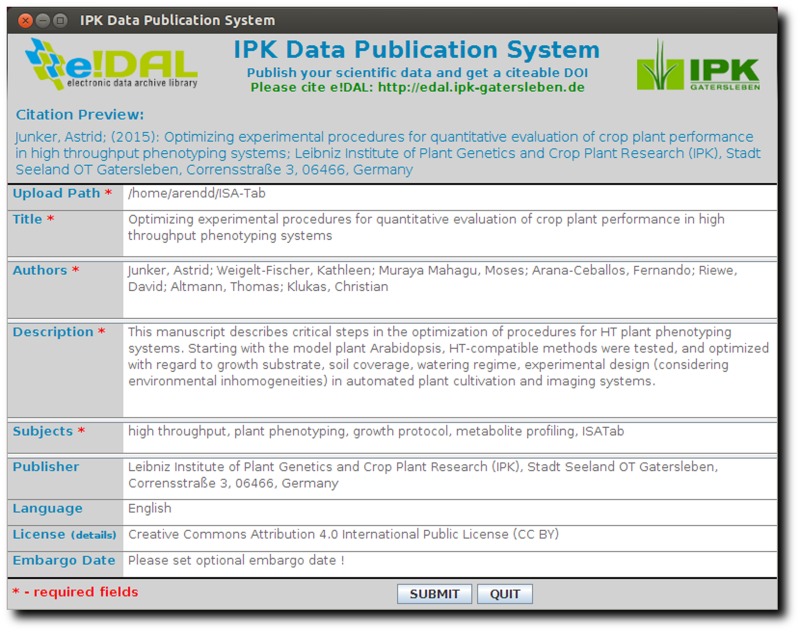
Data publication tool. This screenshot shows the user interface of the submission tool used for data review and publication.

The publication process is initiated by uploading the dataset together with a mandatory subset of technical metadata (see Data submission process section). The metadata, e.g. file size, file type or file format, will be automatically determined. Furthermore, the tool enables to reuse metadata from previous sessions. The screenshot in Figure 6 shows the graphical interface of the publication tool that was developed to submit a dataset and request for a DOI. This platform-independent Java WebStart application was designed to be user-friendly and intuitive. It supports the automatic setup of the network infrastructure, such as proxy and SMTP-server detection.

**Figure 7.**
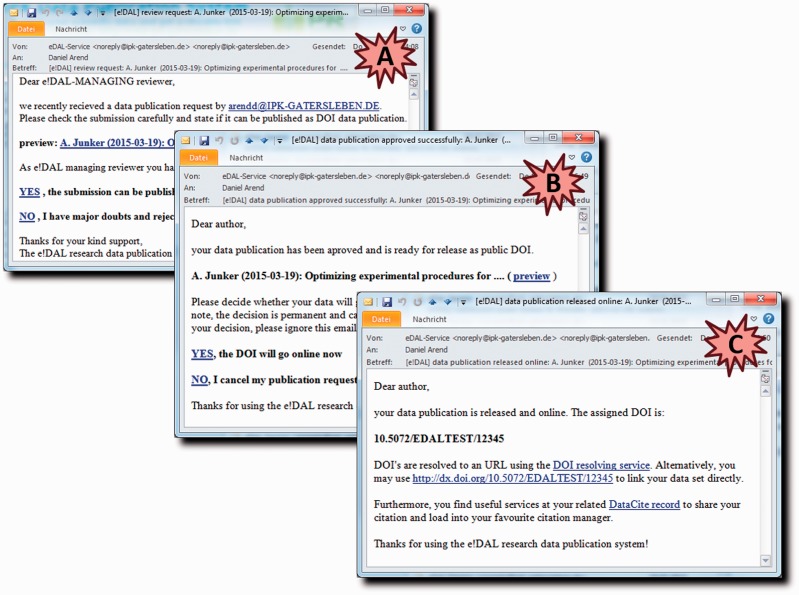
Email notification system. These screenshots show example emails that are generated for communication of the requesting user and the reviewers during the approval process. (**A**) DOI request notification to reviewer. (**B**) Accept notification to requesting user. (**C**) Notification with finally assigned DOI.

In order to facilitate the handling for the user, the graphical user interface was designed in a way that resembles standard submission forms of scientific journals or conferences. An optional embargo date can be defined to hide the public access to DOI landing page of a dataset up to the defined time point. After data upload to the mounted storage backend is finished, the reviewers are notified of the publication request by an email that includes a private URL with restricted access to the submitted files and metadata (Figure 7A).

In case the submission was rejected, the requesting user is informed and asked to revise/re-format the submitted dataset. After successful review, the author is notified (Figure 7B) and can confirm the assigned DOI or alternatively discard the submission. The released DOI will be registered and sent out to the respective user within an additional email (Figure 7C). Alternatively, the author can wait with the final decision to assign a DOI and use the temporary preview link from the notification, e.g. to provide access to the reviewers of a manuscript.

After a new data submission has been recorded, the reviewers immediately receive a notification with private links to data and metadata files, including a request for evaluation (Figure 2). The scientific and administrative reviewers evaluate the dataset with regard to scientific and formal aspects and will give feedback to the author.

#### Approval and DOI registration

In order to approve a data publication and DOI registration, a review workflow containing two scientists and one administrative decision maker was implemented. All reviewers decide whether they accept or reject the request for publishing a dataset. The reviewer’s ratings are combined by a configurable decision matrix to a final decision. The default matrix (Table 1) let a scientific reviewer or its assistant decide for reject or acceptance. The administrative reviewer may confirm their decisions or draw a veto that rejects the entire submission. As mentioned in Data submission process section, the definition of scientific and administrative reviewers increases the data quality and prevent from publishing secret information. Nevertheless, this preconfigured default review process can be customized to reflect other institutional policies.

**Table 1.** Default decision matrix to compute the final reviewing result

Managing reviewer	Senior scientist	Deputy scientist
Accept	Accept	Any
Accept	No response	Accept

**Figure 8.**
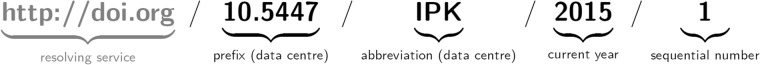
Schema of DOI assignment. This schema used to generate a unique DOI for a new dataset.

For the final DOI registration, it is necessary to transfer the metadata and the access URL of the particular dataset to the DataCite resolving system (http://mds.datacite.org). DataCite provides a REST API to post both as XML document, which has to be conforming to the DataCite Metadata Schema (http://schema.datacite.org). The e!DAL DCMES metadata attributes are mapped to the corresponding DataCite elements. The generation of a unique DOI followed the schema illustrated in Figure 8. The Apache Solr interface of the DataCite Search API (http://search.datacite.org) allows to request the number of existing DOIs, the prefix and name of the registered data centre.

## Summary and outlook

The PGP repository provides access to plant phenomics and genomics research data, which is hitherto not supported by any existing international repository. The main focus of PGP is to publish and share primary experimental data covering a variety of data domains such as image collections from high-throughput plant phenotyping, sequence assemblies, genotyping data, visualizations of morphological plant models, data from mass spectrometry and even software. Datasets in the repository are assigned to citable DOIs that are registered at DataCite with a standardized set of technical metadata.

To lower barriers for researchers in sharing their data, the process of publishing data was made user-friendly and faster and supports best practices for data publication. Those are an online review and notification process, support of embargo and private pre-publication access, bibliographically accepted data citation and the obligation to the scientist to provide a minimal set of widely accepted technical metadata. As result, the data publication has become a self-evident procedure instead of being a handicap for data producers. Currently, PGP provides access to about 22 000 files (http://edal.ipk-gatersleben.de/repos/pgp/). Since January 2015, the repository was accessed by over 1500 unique IPs addresses worldwide. Overall, PGP had in average 2500 clicks per month so far. The downloaded volume is about 82 TB and expresses the demands and interests of the scientific community in primary research data from crop plants.

The integrated Web frontend supports the interactive exploration of available datasets. Beside functionalities to search, filter and download datasets, access matrices are provided for each DOI. The comprehensive API support for programmatic access by DOI-URLs, DataCite APIs, OAI-PMH harvesting protocols and the e!DAL native programming interface enable wide range of bioinformatics tool to search, browse and access the stored datasets. Using those interfaces, we implement the FAIR data paradigm—findable, accessible, interoperable and reusable—for each published dataset. So, the PGP supports the OAI-PMH metadata harvesting protocol, it is registered at BioSharing.org, re3data and OpenAire as valid EU Horizon 2020 ‘open data’ repositories.

Beside the benefits for the public research community, the PGP repository also increases the scientific credit for publishing authors. The reports embedded in PGP for each DOI (dataset) provide statistics about total number of unique downloads of a single DOI, total downloaded data volume per DOI. These statistics are of importance for the author as a personal feedback about the use of submitted datasets and can furthermore can be used to support project proposals since funding agencies increasingly request detailed data management and publication strategies. In addition, high access to specific datasets proves scientific relevance and excellence in the respective field as well (14). More sophisticated data citation metrics, like the Thomson Reuters Data Citation Index (28), are underway to compute impact factors for published datasets, which offer a more consistent impact statistics and complement the common science citation indexes (29). The beforehand mentioned dedicated data journals are a further way to get credit for published experimental data. PGP is accepted as repository under the name ‘IPK Gatersleben’ for the *Nature Scientific Data* journal, so scientist are able to submit along their published dataset a method paper like, the publication of genotyping data from 52 highly diverse accessions of the model allopolyploid plant *Brassica napus* (30).

The e!DAL backend infrastructure is used to set-up and operate the PGP repository. As an extension, we implemented a data submission tool that uses e!DAL’s client-server API and provides an intuitive user interface for data submission, review and publication. The review process was integrated into the e!DAL as novel API functions. The used server backend is a LINUX server with a 4 core CPU and 60 GB RAM. The storage backend is an Oracle Hierarchical Storage Manager with 211 TB storage capacity. As administrative prerequisite to publish DOIs, IPK as PGP hosting institute is registered as data centre.

Future developments on the technical level will comprise the support of cloud-based services, such as distributed service or peer to peer data replication to pin selected PGP datasets to the tool installation and avoid time consuming data downloads. At the level of the datasets, more emphasis will be put to the extension of the currently rather technical metadata towards semantic annotation of published experiments. It is envisaged to supplement more DOIs by ISA-Tab-formatted experiment semantics (including detailed information about experimental conditions, parameters, samples and measurements). In addition, the aim is to integrate data from IPK’s Laboratory Information Management System and PGP repository into a continuous data publication workflow. Thus, an increasing number of sufficiently annotated and well-documented datasets from a variety of data domains will be stored in PGP and thereby made available to the scientific community. In the future, the presented PGP repository might also be opened to allow publication and storage of datasets provided by external users. In its function as a phenomics data repository, PGP is unique and will be further extended in order to support data management and publication in this growing community.

## References

[1] Brooksbank C (2014) The European Bioinformatics Institute's data resources 2014. Nucleic Acids Res., 42(Database issue), D18–D25. 24271396 10.1093/nar/gkt1206 PMC3964968

[2] Craddock T (2008) e-Science: relieving bottlenecks in large-scale genome analyses. Nat. Rev. Microbiol., 6, 948–954. 19008893 10.1038/nrmicro2031

[3] Clarke L (2012) The 1000 Genomes Project: data management and community access. Nat. Methods, 9, 459–462. 22543379 10.1038/nmeth.1974 PMC3340611

[4] Tellam R (2015) The primary reasons behind data sharing, its wider benefits and how to cope with the realities of commercial data. BMC Genom., 16, 1–4. 10.1186/s12864-015-1789-5 PMC4561426 26343138

[5] Chavan V. Penev L. (2011) The data paper: a mechanism to incentivize data publishing in biodiversity science. BMC Bioinform., 12(Suppl 15), S1–S12. 10.1186/1471-2105-12-S15-S2 PMC3287445 22373175

[6] Kodama Y (2012) The Sequence Read Archive: explosive growth of sequencing data. Nucleic Acids Res., 40(Database issue), D54–D56. 22009675 10.1093/nar/gkr854 PMC3245110

[7] Singh J. (2011) FigShare. J. Pharmacol. Pharmacother., 2, 138–139. 21772785 10.4103/0976-500X.81919 PMC3127351

[8] Fernandez-Suarez X.M. Rigden D.J Galperin M.Y. (2014) The 2014 Nucleic Acids Research Database Issue and an updated NAR online Molecular Biology Database Collection. Nucleic Acids Res., 42(Database issue), D1–D6. 24316579 10.1093/nar/gkt1282 PMC3965027

[9] Monaco M.K (2014) Gramene 2013: comparative plant genomics resources. Nucleic Acids Res., 42(Database issue), D1193–D1199. 24217918 10.1093/nar/gkt1110 PMC3964986

[10] Vines T.H (2014) The availability of research data declines rapidly with article age. Curr. Biol., 24, 94–97. 24361065 10.1016/j.cub.2013.11.014

[11] (2009) Data's shameful neglect. Nature, 461, 145. 10.1038/461145a 19741659

[12] Gibney E. Van Noorden R. (2013) Scientists losing data at a rapid rate. Nature, 504 (doi: 10.1038/nature.2013.14416).

[13] Piwowar H.A. (2011) Who shares? Who doesn't? Factors associated with openly archiving raw research data. PLoS One, 6, e18657. 1–13. 21765886 10.1371/journal.pone.0018657 PMC3135593

[14] Piwowar H.A. Day R.S. Fridsma D.B. (2007) Sharing detailed research data is associated with increased citation rate. PLoS One, 2, e308. 1–5. 17375194 10.1371/journal.pone.0000308 PMC1817752

[15] Crosswell L.C. Thornton J.M. (2012) ELIXIR: a distributed infrastructure for European biological data. Trends Biotechnol., 30, 241–242. 22417641 10.1016/j.tibtech.2012.02.002

[16] Lagoze C.H. Van de Sompel (2001) The Open Archives Initiative: Building a low-barrier interoperability framework. In: *Proceedings of the 1st ACM/IEEE-CS Joint Conference on Digital Libraries, 2001*. New York, NY, USA: ACM.

[17] Neumann J. Brase J. (2014) DataCite and DOI names for research data. J. Comput. Aided Mol. Des., 28, 1035–1041. 25038897 10.1007/s10822-014-9776-5

[18] Krajewski P (2015) Towards recommendations for metadata and data handling in plant phenotyping. J. Exp. Bot., 66, 5417–5427. 26044092 10.1093/jxb/erv271

[19] Arend D., (2014) e!DAL–a framework to store, share and publish research data. BMC Bioinform., 15, 1–13. 10.1186/1471-2105-15-214 PMC4080583 24958009

[20] Pampel H (2013) Making Research Data Repositories Visible: The re3data.org Registry. PLoS One, 8, e78080. 1–10. 24223762 10.1371/journal.pone.0078080 PMC3817176

[21] Field D (2009) ‘Omics data sharing. Science, 326, 234–236. 19815759 10.1126/science.1180598 PMC2770171

[22] Sompel H.V (2004) Resource harvesting within the OAI-PMH framework. D-Lib Mag., 10 (http://dspace.library.uu.nl/handle/1874/3163).

[23] Peng R.D. (2011) Reproducible research in computational science. Science, 334, 1226–1227. 22144613 10.1126/science.1213847 PMC3383002

[24] Weibel S. (1997) The Dublin core: a simple content description model for electronic resources. Bull. Am. Soc. Inform. Sci. Technol., 24, 9–11.

[25] Rocca-Serra P (2010) ISA software suite: supporting standards-compliant experimental annotation and enabling curation at the community level. Bioinformatics, 26, 2354–2356. 20679334 10.1093/bioinformatics/btq415 PMC2935443

[26] Neuroth H (2010) *Nestor Handbuch: eine kleine Enzyklopädie der digitalen Langzeitarchivierung v2.3*. Niedersächsische Staats- und Universitätsbibliothek Göttingen, Platz der Göttinger Sieben 1, 37073 Göttingen, Germany.

[27] Field D (2010) Meeting Report: BioSharing at ISMB 2010. Stand. Genom. Sci., 3, 254–258. 10.4056/sigs/1403501 PMC3035313 21304729

[28] Force M.M. Robinson N.J. (2014) Encouraging data citation and discovery with the Data Citation Index. J. Comput. Aided Mol. Des., 28, 1043–1048. 24980647 10.1007/s10822-014-9768-5

[29] Garfield E. (2006) Citation indexes for science. A new dimension in documentation through association of ideas. 1955. Int. J. Epidemiol., 35, 1123–1127, discussion 1127–1128. 16987841 10.1093/ije/dyl189

[30] Schmutzer T (2015) Species-wide genome sequence and nucleotide polymorphisms from the model allopolyploid plant *Brassica napus.* Sci. Data, 2 (doi:10.1038/sdata.2015.72) 10.1038/sdata.2015.72 PMC4672681 26647166

